# Predictors of extra-articular manifestations in axial spondyloarthritis and their influence on TNF-inhibitor prescribing patterns: results from the British Society for Rheumatology Biologics Register in Ankylosing Spondylitis

**DOI:** 10.1136/rmdopen-2020-001206

**Published:** 2020-07-08

**Authors:** Mohammad H Derakhshan, Linda Dean, Gareth T Jones, Stefan Siebert, Karl Gaffney

**Affiliations:** 1 Institute of Infection, Immunity & Inflammation, University of Glasgow, Glasgow, UK; 2 Epidemiology Group, Univesity of Aberdeen, Aberdeen, UK; 3 Rheumatology Department, Norfolk and Norwich University Hospital, Norwich, UK

**Keywords:** Spondyloarthritis, Anti-TNF, Epidemiology

## Abstract

**Objectives:**

Extra-articular manifestations (EAMs) are important systemic features of axial spondyloarthritis (axSpA), which may influence the choice of tumour necrosis factor-inhibitor (TNFi). We examined the cumulative incidence and predictors of EAMs and the influence of these on first TNFi choice in a ‘real-world’ cohort of patients with axSpA.

**Methods:**

Clinical and patient-reported outcomes of 2420 patients with axSpA from 83 centres were collected by the British Society for Rheumatology Biologics Register in Ankylosing Spondylitis. Lifestyle factors for EAMs (acute anterior uveitis (AAU), inflammatory bowel diseases (IBD), psoriasis) were compared with those without EAMs. Also, the association between pretreatment EAMs and choice of first TNFi (adalimumab, etanercept, certolizumab) was analysed.

**Results:**

AAU was directly associated with human leukocyte antigen (HLA)-B27 (incidence rate ratio (IRR) 1.95, 95% CI 1.40 to 2.73) and inversely associated with ever-smoking (IRR=0.71, 95% CI 0.55 to 0.92). For both psoriasis and IBD, there was an inverse relationship with HLA-B27 (IRR 0.54, 95% CI 0.36 to 0.79 and IRR 0.63, 95% CI 0.43 to 0.91, respectively). A diagnosis of either AAU (OR 3.79, 95% CI 2.11 to 6.80) or IBD (OR 5.50, 95% CI 2.09 to 14.46) was associated with preference for adalimumab versus others. In contrast, a diagnosis of either AAU (OR 0.14, 95% CI 0.06 to 0.33) or IBD (OR 0.17, 95% CI 0.05 to 0.57) was associated with less preference for etanercept over other TNFi.

**Conclusion:**

The higher occurrence of AAU and lower occurrence of psoriasis and IBD in HLA-B27-positive patients with axSpA are consistent with current pathophysiology. Patients with previous AAU and IBD are more likely to be prescribed adalimumab and less likely to receive etanercept, consistent with the superior efficacy of monoclonal TNFi for these indications. Future work will determine whether EAMs influence TNFi survival, or effectiveness, and whether this varies between agents.

## INTRODUCTION

Extra-articular manifestations (EAMs) are common and important systemic features of axial spondyloarthritis (axSpA), the most common being acute anterior uveitis (AAU), inflammatory bowel disease (IBD) and psoriasis. It has previously been estimated that the pooled prevalence of AAU, psoriasis and IBD in axSpA are 16–23%, 10–11% and 4–6%, respectively.^1^ The prevalence of EAMs is broadly similar in ankylosing spondylitis (AS; also called radiographic axSpA) and non-radiographic axSpA (nr-axSpA), except for AAU prevalence that is higher in AS than nr-axSpA.^1^ EAMs contribute to the disease burden and add a layer of complexity to the management of axSpA.^2 3^

National and international recommendations for the management of axSpA consistently highlight the importance of considering EAMs when choosing the appropriate tumour necrosis factor inhibitor (TNFi) due to their different modes of action and efficacy in the context of individual EAMs.^2 4 5^ In the UK, Europe and North America, five originator TNFi are currently licensed to treat axSpA; adalimumab, certolizumab pegol, etanercept, golimumab and infliximab (AS only). Several TNFi are also licensed to treat psoriasis (all except golimumab) and IBD (ulcerative colitis—adalimumab, infliximab and golimumab; Crohn’s disease—adalimumab and infliximab (certolizumab in the USA and Switzerland only)). However, no TNFi are currently licensed to treat AAU in adults, although adalimumab is licensed to treat non-infectious intermediate, posterior and panuveitis.^6^ In a post hoc analysis of randomised controlled trials for axSpA and other conditions, as well as in many observational studies, it has been shown that monoclonal TNFi are efficacious in AAU, with monoclonal antibody TNFi showing superior efficacy over etanercept.^7–^^9^

EAMs therefore have important implications for the use of TNFi, particularly the soluble fusion protein etanercept, in the management of axSpA. However, it is not feasible or ethical to randomise patients with axSpA and specific EAMs to specific treatments in conventional randomised controlled trial settings, so data from real-world cohorts are required. Specifically, physicians’ beliefs and knowledge about the efficacy and safety of specific TNFi for AAU, IBD and psoriasis are likely to influence their choice of TNFi in patients with axSpA. The purpose of this study is to examine the cumulative incidence and predictors for developing EAMs and their influence on physicians’ prescribing patterns for the first TNFi in a ‘real-life’ cohort of patients with axSpA recruited in the UK.

## METHODS

### Participants data

The British Society for Rheumatology Biologics Register in Ankylosing Spondylitis (BSRBR-AS) is a UK-wide prospective observational cohort study of patients with axSpA recruited from 83 centres and fulfilling the Assessment of SpondyloArthritis International Society (ASAS) classification criteria.^10^ The BSRBR-AS study protocol has been published previously.^11^ Baseline data were obtained between December 2012 and June 2017 using a combination of a targeted medical history, participant self-completed questionnaires and the participants’ medical records. The presence or absence of EAMs (ever diagnosis), defined as physician-verified diagnosis of AAU, psoriasis and IBD, were specifically recorded prior to commencing biological therapy. Demographic data included age, disease duration (time between symptom onset to BSRBRAS baseline visit), body mass index (BMI), cigarette smoking status (ever vs never used for this analysis), alcohol consumption and educational status. Human leukocyte antigen (HLA)-B27 status was determined from either medical records or analysis of patients’ saliva samples.

In the original data set, there were only a small proportion of missing data (<5%) in variables of interests; therefore, we have not applied any imputation methods to our data set.

Ethical approval was obtained from the National Research Ethics Service Committee North East—County Durham and Tees Valley (reference 11/NE/0374), and signed informed consent was obtained from all participants.

### Statistical analysis

Descriptive statistics were used to compare participant characteristics according to presence or absence (ever diagnosis) of AAU, psoriasis and IBD (either Crohn’s disease or ulcerative colitis), with central tendencies in groups presented as median and IQR, unless otherwise stated. Where necessary, the difference between independent groups was examined using the Mann-Whitney U test. Poisson log-linear models were used to examine the associations between each EAM (AAU, psoriasis, IBD) and demographic, lifestyle and HLA-B27 status. In unadjusted models, estimates of incidence rate ratio (IRR) and relevant 95% CI were calculated with one EAM as response and one factor as predictor. In adjusted models, all factors (categorical) and covariate (continuous) variables showing significant association (p<0.05) with the response in univariable analysis were entered in the model. In final models, when AAU was the response, HLA-B27 (reference: negative), university degree (reference: no) and ever-smoking (reference: never) were factors, and age (years) and axSpA disease duration (years) were covariates. In the model for psoriasis, HLA-B27 (reference: negative), university degree (reference: no) and ever-smoking (reference: never) were factors, and age (years) and BMI were covariates. In the model for IBD as response, only HLA-B27 (reference: negative) was entered as factor, and age (years) and axSpA disease duration (years) as covariates. For each EAM, the final models’ type was Poisson log-linear, the maximum iterations were 100 and maximum step-halving was 5. The convergence criteria defined as a change in parameter estimate of 1e-006 or more.

To examine the associations between EAMs and the choice of first TNFi, logistic regression models were used. In univariable analyses, separate models were constructed with etanercept versus monoclonal TNFis as dependent variable, and pretreatment diagnosis of AAU, psoriasis and IBD (as Crohn’s disease only, ulcerative colitis only and combined). Other demographic and lifestyle factors included in the models were age, gender, axSpA disease duration, BASDAI (Bath Ankylosing Spondylitis Disease Activity Index) score, BASFI (Bath Ankylosing Spondylitis Functional Index) score, HLA-B27 status, smoking status, education and BMI. Factors with significant associations (p<0.05) in univariable models were entered into the multivariable model, again with choice of TNFi (etanercept vs monoclonal TNFis) as dependent variable. Pretreatment diagnosis of AAU, IBD, education status, BASDAI and BASFI were included in the final model. The entered method was chosen for the final multivariable analyses to keep all variable in the model. Magnitude of associations were presented as ORs and 95% CI.

## RESULTS

Baseline data for 2419 participants were available for the current analysis. Approximately a third of the cohort (927; 32%) had one or more EAM of interest present prior to receiving biological therapy; of these 780 (84.1%) had only one EAM, 142 (15.3%) had two EAMs and 5 (0.5%) participants had all three EAMs. The frequency of AAU, psoriasis and IBD at the baseline visit were 23.5% (568), 10.9% (264) and 10.2% (247), respectively. The baseline demographic, lifestyle factors and HLA-B27 status of the whole cohort and those with EAMs are shown in table 1.

**Table 1:** Characteristics of entire cohort and subgroups with extra-articular manifestations

	Entire cohort (n=2419)	With AAU (n=568, 23.5%)	With psoriasis (n=264, 10.9%)	With IBD (n=247, 10.2%)
Gender (male)	68.4%	66.7%	67.4%	65.2%
Age (years), median (IQR)	47.0 (36.0–59.0)	51.0 (41.0–61.0)	51.5 (38–62)	52.0 (40.0–63.0)
AxSpA disease duration (years), median (IQR)	16.0 (7.0–30.0)	22.0 (11.0–34.0)	17.5 (7.0–32.0)	20.0 (8.0–35.0)
BMI (kg/m^2^), median (IQR)	26.8 (23.9–30.7)	27.3 (24.0–31.0)	27.7 (24.4–31.4)	27.1 (24.0–30.8)
HLA-B27 positive	80.1%	88.7%	66.7%	71.4%
University degree	27.8%	33.5%	27.2%	24.8%
Ever-smoker	56.2%	50.1%	62.7%	59.7%
Ever-drinking alcohol	93.0%	94.2%	94.0%	92.1%

AAU, acute anterior uveitis; axSpA, axial spondyloarthritis; BMI, body mass index; HLA, human leukocyte antigen; IBD, inflammatory bowel disease.

### Associations with AAU

AAU was the most common EAM reported in 568 (23.5%) of participants (table 1). In the univariable analysis, significant positive associations in the AAU cohort included older age (IRR 1.01, 95% CI 1.01 to 1.021 per year), longer disease duration (IRR 1.02, 95% CI 1.01 to 1.02), HLA-B27 (IRR 1.92, 95% CI 1.42 to 2.61) and university education (1.30, 95% CI 1.08 to 1.57). Conversely, the proportion of patients who ever-smoked was significantly lower in the AAU group (IRR 0.77, 95% CI 0.64 to 0.92). Additional analysis revealed a confounding between education and ever-smoking, indicating that the higher occurrence of AAU in those with university education was due to lower proportion of ever-smoking in that group (ever-smoking was 20.9% in those with university degree compared to 79.1% in those without a university degree; p<0.001). There was no association between AAU and gender, BMI or alcohol consumption.

In the multivariable analysis (table 2), HLA-B27 was associated with an increase in the risk of AAU (IRR 1.95, 95% CI 1.40 to 2.73). In contrast, ever-smoking was associated with reduced risk of AAU (IRR 0.71, 95% CI 0.55 to 0.92). Other parameters including university education, age and axSpA disease duration did not demonstrate a significant association with AAU in this cohort using our predefined cut-offs, although age and disease duration were assessed per year so may still have a cumulative effect over time.

**Table 2:** Factors and covariates associated with a diagnosis of specific EAM in patients with axSpA (multivariable models)

Parameter	B	SE	P value	Incidence rate ratio (IRR)	95% CI for IRR
A. Diagnosis of AUU					
(Intercept)	−2.370	0.2788	<0.001	0.093	0.054–0.161
HLA-B27 positive (vs negative)	0.669	0.1710	<0.001	1.953	1.396–2.730
University degree (vs lower)	0.165	0.1507	0.273	1.179	0.878–1.585
Ever-smoking (vs never)	−0.349	0.1322	0.008	0.706	0.545–0.915
Age (years)	0.009	0.0053	0.093	1.009	0.999–1.019
axSpA disease duration (years)	0.005	0.0050	0.305	1.005	0.995–1.015
B. Diagnosis of Psoriasis					
(Intercept)	−3.069	0.592	0.000	0.046	0.015–0.148
HLA-B27 positive (vs negative)	−0.621	0.197	0.002	0.538	0.365–0.791
University degree (vs lower)	0.326	0.283	0.248	1.386	0.796–2.412
Ever-smoking (vs never)	0.256	0.228	0.262	1.292	0.826–2.020
Age (years)	0.009	0.007	0.173	1.009	0.996–1.022
BMI (kg/m^2^)	0.022	0.016	0.155	1.023	0.992–1.05
C. Diagnosis of IBD					
(Intercept)	−2.698	0.3617	<0.001	0.067	0.033–0.137
HLA-B27 positive (vs negative)	−0.466	0.1894	0.014	0.627	0.433–0.909
Age (years)	0.012	0.0079	0.128	1.012	0.997–1.028
axSpA disease duration (years)	0.003	0.0078	0.671	1.003	0.988–1.019

AAU, acute anterior uveitis; axSpA, axial spondyloarthritis; BMI, body mass index; EAM, extra-articular manifestation; HLA, human leukocyte antigen; IBD, inflammatory bowel disease.

### Associations with psoriasis

Psoriasis was reported in 264 (10.9%). In univariable analysis, significant positive associations with psoriasis were seen for older age (IRR 1.01, 95% CI 1.01 to 1.02), BMI (IRR 1.03, 95% CI 1.01 to 1.05) and ever-smoking (IRR 1.30, 95% CI 1.00 to 1.70), while the participants with axSpA and psoriasis were less likely to be HLA-B27 positive (IRR 0.50, 95% CI 0.36 to 0.64). Ever-drinking alcohol or having a university degree were not significantly different in the psoriasis group compared to the entire cohort. In the multivariable analysis (table 2), only HLA-B27 status remained as independent predictor of psoriasis (IRR 0.54, 95% CI 0.37 to 0.79). The other parameters, including age and BMI, did not demonstrate a statistically significant association with a diagnosis of psoriasis in this axSpA cohort.

### Associations with IBD

There were 247 (10.2%) patients with a diagnosis of IBD (table 1). In the univariable analysis, positive associations with IBD were older age (IRR 1.02, 95% CI 1.01 to 1.03) and longer axSpA disease duration (IRR 1.01, 95% CI 1.00 to 1.02). In contrast, HLA-B27 was significantly lower in the IBD group (IRR 0.65, 95% CI 0.43 to 0.89). There were no significant associations in this cohort between a diagnosis of IBD and ever-smoking, ever-drinking, BMI or education level.

Similar to the findings in participants with psoriasis, multivariable analysis identified only HLA-B27 status as an independent negative predictor of a diagnosis of IBD with an IRR of 0.63 (95% CI 0.43 to 0.91). Other parameters including age and axSpA disease duration did not demonstrate a statistically significant association with IBD (table 2).

### EAMs and choice of biologic

A total of 947 (39.1%) participants received their first biologic during the course of the observation period. The TNFi biologics were adalimumab (n=598, 63.1%; all Humira), etanercept (n=266, 28.1%; consisted of 240 Enbrel and 26 biosimilar Benepali), certolizumab (n=73, 7.7%) and golimumab (n=9, 1.0%). One participant (0.1%) treated with the interleukin (IL)-17A inhibitor, secukimumab, was excluded from this analysis. Overall, the presence of EAMs did not influence the likelihood of receiving treatment with TNFi, with 66% of participants with an EAM receiving TNFi compared with 66.4% without an EAM (p=0.836).

Treatment and choice of biologic in the BSRBR-AS cohort was at the discretion of the usual treating clinical physician and unrelated to participation in the study. In order to determine which demographic factors and disease measurements were associated with selection of the soluble fusion protein TNFi, etanercept, compared to the monoclonal antibody TNFis (adalimumab, certolizumab or golimumab in this cohort), separate logistic regression models were developed. Univariable analysis revealed that baseline BASDAI score (OR 1.15, 95% CI 1.06 to 1.24) and BASFI score (OR 1.11, 95% CI 1.04 to 1.18) were significantly associated with the choice of etanercept over monoclonal TNFi. Conversely, having higher education (university education and above) was associated with less use of etanercept over monoclonal TNFi (OR 0.73, 95% CI 0.54 to 0.99). None of other demographic or lifestyle variables, including age, gender, disease duration, HLA-B27, smoking and BMI, were associated with the choice of TNFi.

Considering EAMs in the univariable models, ever diagnosis of AAU (as binary yes: no) and number of AAU episodes in the last 12 months were both significantly associated with less use of etanercept compared to monoclonal TNFi (OR 0.29, 95% CI 0.19 to 0.46 and OR 0.48, 95% CI 0.30 to 0.77, respectively)). Also, patients with ever diagnosis of IBD in any form (all IBD, Crohn’s disease alone and ulcerative colitis alone) were less likely to receive etanercept over monoclonal TNFi in univariable models (OR 0.35, 95% CI 0.20 to 0.62). In contrast, ever diagnosis of psoriasis was not associated with choice of any particular TNFi (OR 0.74, 95% CI 0.47 to 1.16) (table 3).

In multivariable analysis (table 4), both ever diagnosis of AAU (OR 0.15, 95% CI 0.08 to 0.29) and ever diagnosis of IBD (OR 0.30, 95% CI 0.16 to 0.56) remained significantly associated with less use of etanercept compared to monoclonal TNFis. Among other factors, only BASDAI score had a significant association with the preference of etanercept over monoclonal TNFi (OR 1.16, 95% CI 1.03 to 1.30).

**Table 3:** Summary of factors associated with choice of etanercept over monoclonal antibody TNFis in patients with axSpA (summary of separate univariable models)

Variable	Wald	P value	OR	95% CI for OR
Gender (male vs female)	3.561	0.059	1.351	0.988–1.847
Age (years)	0.842	0.359	0.995	0.984–1.006
Disease duration (years)	2.062	0.151	0.992	0.980–1.003
BASDAI score	12.226	<0.001	1.146	1.062–1.237
BASFI score	10.393	0.001	1.107	1.041–1.178
HLA-B27 positivity	0.033	0.856	1.038	0.692–1.557
Smoking (current vs none)	1.525	0.217	1.268	0.870–1.848
Smoking (ex-smoking vs none)	0.084	0.772	1.054	0.736–1.510
Education (ordinal)	3.543	0.060	0.893	0.793–1.005
Education (university degree vs lower)	4.084	0.043	0.730	0.539–0.991
BMI (kg/m^2^)	0.365	0.546	0.956	0.826–1.106
AAU (any)	27.943	<0.001	0.294	0.187–0.463
AAU episodes in last 12 months	9.336	0.002	0.484	0.304–0.771)
IBD (Crohn’s disease or ulcerative colitis)	13.081	<0.001	0.354	0.201–0.621
Crohn’s disease	9.457	0.002	0.044	0.006–0.323
Ulcerative colitis	7.456	0.006	0.192	0.056–0.628
Psoriasis	1.707	0.191	0.738	0.468–1.164

AAU, acute anterior uveitis; axSpA, axial spondyloarthritis; BASDAI, Bath Ankylosing Spondylitis Disease Activity Index; BASFI, Bath Ankylosing Spondylitis Functional Index; BMI, body mass index; EAM, extra-articular manifestation; HLA, human leukocyte antigen; IBD, inflammatory bowel disease; TNFi, tumour necrosis factor-inhibitor.

**Table 4:** Summary of factors associated with choice of etanercept over monoclonal TNFis in patients wth axSpA (summary of multivariable model)

Variable	Wald	P value	OR	95% CI for OR
BASDAI score	5.941	0.015	1.158	1.029–1.304
BASFI score	0.315	0.577	1.028	0.932–1.135
Education (university degree vs lower)	1.098	0.295	0.836	0.597–1.169
AAU (any)	35.266	<0.001	0.154	0.083–0.286
IBD (Crohn’s disease or ulcerative colitis)	13.853	<0.001	0.296	0.156–0.562

AAU, acute anterior uveitis; axSpA, axial spondyloarthritis; BASDAI, Bath Ankylosing Spondylitis Disease Activity Index; BASFI, Bath Ankylosing Spondylitis Functional Index; IBD, inflammatory bowel disease; TNFi, tumour necrosis factor-inhibitor.

## DISCUSSION

In this large real-world cohort of patients with axSpA, EAMs are common, with approximately a third of participants reporting at least one EAM. The occurrence of EAMs in this cohort is approximately similar to previous published reports in similar populations.^12–^^14^ Interestingly, among patients who had EAMs, most (84%) of the participants only had a single EAM, suggesting that while there may be shared pathogenetic processes between EAMs and axSpA, there are also likely to be unrelated pathogenic mechanisms. This is consistent with the existing and emerging pathogenetic and treatment response data for these conditions.^15 16^

The presence of AAU in this axSpA cohort was significantly associated with HLA-B27 status. This is consistent with previous reports^7 17^ and offers a potential strategy for targeted identification of people with AAU in ophthalmology clinics who may have underlying axSpA.^18–^^20^ Interestingly, participants with IBD and psoriasis had lower rates of HLA-B27 positivity than the overall cohort and both had negative associations with HLA-B27 status, consistent with results from a recent study by Varkas *et al.*^21^ This may indicate that people with psoriasis or IBD and back pain are more likely to be diagnosed with axSpA, regardless of their HLA-B27 status. Interestingly, a positive HLA-B27 (plus at least two other SpA features, which could include psoriasis or IBD) is required to fulfil the clinical arm of the ASAS classification criteria for axSpA^10^; this may suggest that these participants are more likely to fulfil the imaging arm (HLA-B27 not required). An alternative explanation could be that clinicians rely more on diagnostic likelihood and clinical acumen rather than rigorously adhering to classification criteria, which would be appropriate as classification criteria are not intended for diagnostic use in clinical practice.^22 23^ Finally, it is also possible that axSpA is under-diagnosed in patients with dominant IBD or psoriasis, particularly those who are HLA-B27 positive.^24–^^27^

The current study indicated that smoking is associated with lower occurrence of AAU but not associated with psoriasis or IBD. The inverse association between AAU and smoking was evident in both unadjusted and adjusted models. However, smoking is considered an established risk factor for AAU^28^ and its negative association with AAU in this study should be interpreted with caution. First, there are differences between studies regarding comparison groups; all participants of the current study had confirmed axSpA, in contrast to studies reporting the associations with different background diseases or even in the general population.^29^ Furthermore, while a similar inverse association between smoking and AAU was reported in a recent study using the same registry data,^30^ subsequent analysis showed that smoking does not protect patients with axSpA from uveitis attacks.^31^ Other studies have also demonstrated this negative association between smoking and uveitis: Jones *et al*, in a study of around 1000 patients from Scotland, found that ex-smokers were more than twice as likely to have a history of uveitis compared to current smokers.^32^ Further investigation using different populations and methodology designed specifically to examine the relationship between AAU and smoking in patients with axSpA is required.^33^

The presence of EAMs in general did not increase the likelihood of participants being started on a biologic in this cohort. This presumably reflects the focus on axSpA disease activity threshold scores in the axSpA treatment recommendations,^2 4 5^ such that the decision to prescribe biologics in axSpA is based largely on axSpA disease activity assessment by the rheumatology team, rather than the overall disease burden. IBD and psoriasis often require biological therapy in their own right (prescribed by gastroenterologists or dermatologists), but these patients are unlikely to be captured in this cohort which only recruited participants from rheumatology clinics, so the cohort may have missed those with the most severe IBD, psoriasis or AAU. As EAMs contribute to the disease burden in axSpA,^2 3^ it could be argued that a composite axSpA disease burden score incorporating EAMs, and functional measures, might be more representative of the true axSpA disease burden than the current disease activity scores (BASDAI and ASDAS), which are dominated by subjective pain and fatigue scores. This approach may be worthy of further study and help address the value of combined clinics with other specialties. Although we believe that rheumatologists usually take the full burden of disease into consideration, in many countries, patient-reported outcomes are used as a reimbursement criterion.

The presence of EAMs, specifically IBD and AAU, did however influence the choice of TNFi. It is clear that rheumatologists are taking the lack of evidence (or even paradox effect) for etanercept in IBD, and to a lesser extent also AAU^34^ into consideration when choosing a specific TNFi for patients with axSpA. The presence of psoriasis did not appear to influence the choice of TNFi in this cohort, which was largely recruited prior to the introduction and wide-spread use of IL-17A inhibitors. EAMs are likely to have a major impact on the choice of TNFi versus IL-17A inhibitors in axSpA as there remains concern about the latter exacerbating IBD.^35 36^ In contrast, IL-17A inhibitors have been shown in head-to-head studies to have greater efficacy in psoriasis.^37 38^ It should be noted that to-date no head-to-head studies of biologics have been reported in axSpA, so data from observational studies will be required to help address these questions. It is unfortunate that, due to the funding of the BSRBR-AS, for the majority of the data collection period only certain TNFi agents were eligible for inclusion. For example, the study therefore has no participants commencing infliximab as first-choice TNFi. However, in the current analysis, all monoclonal antibodies were analysed as a group, and we believe it is unlikely that the additional of patients on infliximab (had that data been available) would have altered the conclusions in the comparison between monoclonal antibodies versus the soluble fusion protein etanercept. Beyond the impact of EAMs on choice of biologic in axSpA assessed in this study, longitudinal real-world studies will be required to assess the impact of EAMs on TNFi retention, which will help inform treatment decisions and recommendations for the use of biologics in axSpA. Lindstrom *et al* reported that AAU and psoriasis but not IBD affect retention of first TNFi in a Swedish cohort of patients with AS^39^; however, more data are needed.

This ‘real-world’ cohort provides valuable information on the occurrence of EAMs in axSpA and their influence on TNFi prescribing patterns. The main strength of the study is the large sample size and the fact that patients have been recruited from 83 specialist and non-specialist centres throughout the UK. To our knowledge, this is the first study designed to address this subject. It is reassuring to note that clinicians are prescribing in line with licenced and international recommendations. Future analysis will determine whether EAMs influence TNF efficacy and survival and whether or not individual TNFi protect patients from incident or flares of existing EAMs.

**Key messages**

**What is already known about this subject**

- Extra-articular manifestations (EAMs) are common and important features of axial spondyloarthritis (axSpA).
- The efficacy of TNF-inhibitors (TNFis) for EAMs is variable due to their different modes of action.

**What this study add**

- This study demonstrates that one in three patients with axSpA have at least one EAM; however, 84% have a single EAM, suggesting that there are unrelated pathogenic mechanisms.
- Acute anterior uveitis is significantly associated with HLA-B27; however, patients with psoriasis and inflammatory bowel disease have lower rates of HLA-B27 positivity than the overall cohort.
- The presence of EAMs does not increase the likelihood of patients being started on a TNFi; however, EAMs do influence TNFi choice.

**How might this impact on clinical practice**

- This study raises awareness of potential impact of EAMs on prescribing patterns in axSpA. Longitudinal analysis will determine whether EAMs influence TNFi survival and whether or not individual TNFi protect patients from incident or flares of existing EAMs.
